# The breeding strategy of female jumbo squid *Dosidicus gigas*: energy acquisition and allocation

**DOI:** 10.1038/s41598-020-66703-5

**Published:** 2020-06-15

**Authors:** Xinjun Chen, Fei Han, Kai Zhu, André E. Punt, Dongming Lin

**Affiliations:** 1 College of Marine Sciences, Shanghai Ocean University, Shanghai, 201306 China; 2 Key Laboratory of Sustainable Exploitation of Oceanic Fisheries Resources, Ministry of Education, Shanghai, 201306 China; 3 National Engineering Research Center for Oceanic Fisheries, Ministry of Science and Technology, Shanghai, 201306 China; 4 Key Laboratory of Oceanic Fisheries Exploration, Ministry of Agriculture and Rural Affairs, Shanghai, 201306 China; 5 Scientific Observing and Experimental Station of Oceanic Fishery Resources, Ministry of Agriculture and Rural Affairs, Shanghai, 201306 China; 6 Laboratory for Marine Fisheries Science and Food Production Processes, Qingdao National Laboratory for Marine Science and Technology, Qingdao, 266071 China; 7 School of Aquatic and Fishery Sciences, University of Washington, Seattle, WA 98195-5020 USA

**Keywords:** Animal behaviour, Marine biology, Ecosystem ecology

## Abstract

Reproductive investment generally involves a trade-off between somatic growth and energy allocation for reproduction. Previous studies have inferred that jumbo squid *Dosidicus gigas* support growth during maturation through continuous feeding (an “income” source). However, our recent work suggests possible remobilization of soma during maturation (a “capital” source). We used fatty acids as biochemical indicators to investigate energy acquisition and allocation to reproduction for female *D. gigas*. We compared the fatty acid profiles of the ovary to those of the mantle muscle (slow turnover rate tissue, representing an energy reserve) and the digestive gland (fast turnover rate organ, reflecting recent consumption). For each tissue, the overall fatty acids among maturity stages overlapped and were similar. The changes with maturation in fatty acid composition in the ovary consistently resembled those of the digestive gland, with the similarity of fatty acids in the mantle muscle and the ovary increasing during maturation, indicating some energy reserves were utilized. Additionally, squid maintained body condition during maturation regardless of increasing investment in reproduction and a decline in feeding intensity. Cumulatively, *D. gigas* adopt a mixed income-capital breeding strategy in that energy for reproduction is mainly derived from direct food intake, but there is limited somatic reserve remobilization.

## Introduction

Life-history theory predicts that individuals should trade-off energy allocation between reproduction and somatic growth or even survival to maximize lifetime reproductive success^1^. Squids are characterized by short lifespan, fast growth and considerable flexibility in reproductive characteristics^2,3^. Although reproduction is typically semelparous, some species spawn multiple times and others continuously^2^, and reproductive behavior relates to how energy is allocated to reproduction during maturation^4–7^. For example, the deep-sea squid *Onykia ingens* reduces somatic growth by utilizing mantle muscle as an energy source to fuel reproduction, which then results in ovarian development for a terminal spawning event^6^. In contrast, the purpleback squid *Sthenoteuthis oualaniensis* apparently supports reproduction using energy acquired directly from food intake, leading to multiple spawning events and continuous growth before death^4^. The former is referred to as a capital breeder, and the latter as an income breeder^8^. Since how energy is allocated during life is central to life-history theory^1^, an optimal trade-off between investment in reproduction and somatic growth has been found to maximize reproductive success, and ultimately determine population size and stability over time^9–11^.

The jumbo squid, *Dosidicus gigas* is one of the most abundant nektonic squid in the eastern Pacific^12^, as well as the target species of major cephalopod fisheries^13^. It plays an important role in pelagic ecosystems locally^14^, not only because it preys on a wide spectrum of organisms during ontogenesis, but also because it is prey for other predators, including marine mammals^15,16^. Similar to other squid species, *D. gigas* is short-lived, usually 1–2 years^17,18^, with a single reproductive episode and multiple spawning events^19–21^. *D. gigas* also responds to environmental conditions^22,23^, which influences how it invests in reproductive development, and hence annual variation of recruitment biomass^24,25^. Rocha *et al*.^2^, Nigmatullin and Markaida^19^ and Hernández-Muñoz *et al*.^20^ suggested that energy allocation to reproduction in *D. gigas* is directly derived from the intake of food, evidenced by the multiple spawning events, non-stop feeding and somatic growth in adults between egg batches. However, recent work by Han *et al*.^26^ on body condition and reproductive investment, involving estimates of the gonadosomatic index and the residuals of a regression of gonad weight on mantle length, suggested that *D. gigas* may also use energy reserves to support reproductive growth.

Fatty acid analyses have been used widely to infer dietary history and trophic ecology for marine species^27–29^, and to some extent, to provide information on how energy is acquired and allocated to tissue types^30,31^. In marine environments, many fatty acids, particularly polyunsaturated fatty acids, can be biosynthesized by certain phytoplankton and microalgae species^32^. In contrast, marine animals are subject to biochemical limitations in biosynthesis and modification of fatty acids, and directly assimilate dietary fatty acids in their basic form without modification^33–36^. In cephalopods, the digestive gland is important for digestion and absorption^37,38^, and has a fast turnover of dietary fatty acids, reflecting more recent food intake (10–14 days^39,40^), and is hence considered a good indicator of nutritional status due to the high lipid concentration^41^. In contrast, tissues such as the mantle muscle are considered the most important energy reserve, with a slower fatty acid turnover rate, that reflects diet over a longer period of time (~4 weeks or longer^39^). Thus, whether gonads are formed using income sources or energy stored in the somatic tissues can be evaluated by comparing fatty acid profiles of these fast and slow turnover tissues with those of the gonads^42^. This comparison could also reveal whether individuals change feeding habits with maturation to obtain greater energy for reproduction^43^.

Following Lin *et al*.^42^ who used fatty acids as biomarkers to determine the mixed income-capital breeding strategy for the female Argentinean shortfin squid *Illex argentinus*, we used fatty acids to investigate the breeding strategy of female *D. gigas* with respect to energy acquisition and allocation. More specifically, we analyzed fatty acids in the digestive gland, the mantle muscle and the ovary to (a) assess whether *D. gigas* shifts its diet to acquire more energy with maturation, (b) determine the pathway of energy sources for reproduction, and (c) justify whether the energy reserve in the somatic tissues are used for reproduction. The results of this work will lead to a better understanding of the breeding strategy of *D. gigas*, and also further support the use of fatty acids to study energy allocation to reproduction for oceanic squid and other species.

## Results

### Fatty acids within tissues

Twenty-eight fatty acids were found in female *D. gigas*, of which 19 had relative mean values greater than 0.5% and in total made up 92–98% of total fatty acids (Table 1). For each tissue, most of the saturated fatty acid (SFA) content was 16:0 and 18:0, most of the monosaturated fatty acid (MUFA) content 18:1n9c and 20:1, and most of the PUFA content 20:5n3 and 22:6n3 (Table 1). The total fatty acid content was higher for functionally mature animals in all tissues analysed, with the highest values consistently observed in the digestive gland (Supplementary Tables 1–3).

**Table 1:** Fatty acid composition in the ovary, the mantle muscle and the digestive gland of female *Dosidicus gigas*.

Fatty acid	Ovary	Mantle	Digestive gland
*Fatty acid (%* total FAs)			
14:0	1.90 ± 1.79	0.77 ± 0.10	4.49 ± 1.42
15:0	0.47 ± 0.29	0.49 ± 0.09	1.02 ± 0.50
16:0	13.45 ± 9.13	22.72 ± 2.04	24.55 ± 6.56
17:0	1.80 ± 1.39	1.00 ± 0.13	1.69 ± 0.78
18:0	9.16 ± 5.10	5.83 ± 0.51	9.07 ± 2.03
20:0	0.31 ± 0.20	0.25 ± 0.06	0.50 ± 0.17
SAF	28.63 ± 6.98	32.80 ± 2.24	42.45 ± 10.38
16:1n7	0.49 ± 0.18	0.34 ± 0.06	3.82 ± 1.98
17:1n7	0.83 ± 2.62	0.03 ± 0.06	1.13 ± 0.49
18:1n9c	3.81 ± 3.10	1.56 ± 0.25	7.79 ± 7.40
20:1	12.28 ± 2.84	6.06 ± 1.08	6.08 ± 2.78
22:1n9	0.36 ± 0.13	0.22 ± 0.04	0.50 ± 0.30
24:1n9	0.33 ± 0.11	0.18 ± 0.04	1.13 ± 0.36
MUFA	18.83 ± 4.17	8.84 ± 1.12	20.79 ± 6.37
18:2n6c	0.32 ± 0.17	0.16 ± 0.07	1.01 ± 0.35
20:2	0.85 ± 0.33	0.37 ± 0.09	1.34 ± 0.61
20:3n3	3.67 ± 1.39	0.95 ± 0.61	0.93 ± 0.75
20:4n6(ARA)	5.42 ± 1.53	2.08 ± 0.41	3.92 ± 1.47
22:2n6	0.49 ± 1.25	0.15 ± 0.09	0.14 ± 0.04
20:5n3(EPA)	16.59 ± 8.47	11.90 ± 1.32	8.71 ± 3.74
22:6n3(DHA)	24.63 ± 8.59	42.26 ± 1.45	19.75 ± 10.94
PUFA	52.54 ± 7.42	58.35 ± 1.62	36.76 ± 11.59
∑FAs<0.5%	4.63 ± 2.33	4.36 ± 1.06	2.57 ± 0.49
*Total fatty acids (mg/g dry weight)*			
total FAs	107.46 ± 47.98	95.25 ± 37.91	234.82 ± 111.33

FAs <0.5% include 15:0, 20:0, 16:1n7, 17:1n7, 22:1n9, 24:1n9, 18:2n6c, 20:2, 22:2n6. ARA, arachidonic acid; EPA, eicosapentaenoic acid; DHA, docosahexaenoic acid; SFA, saturated fatty acids; MUFA, monounsaturated fatty acids; PUFA, polyunsaturated fatty acids; total FAs, total fatty acids. Values are mean ± standard deviation; total FAs is reported as dry tissue weight (mg/g dry weight), other values are reported as percentages of total FAs (% total FAs).

**Figure 1:**
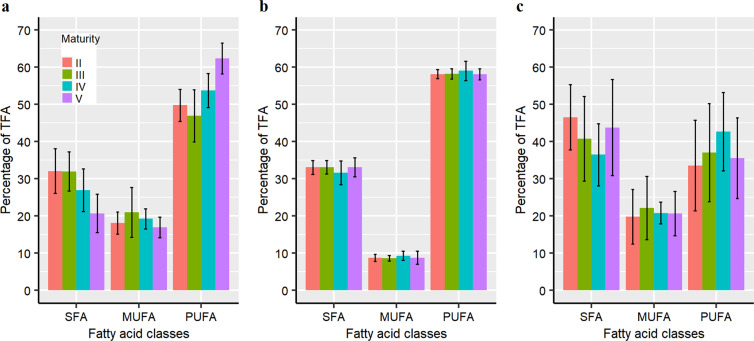
The relative content of saturated fatty acids (SFA), monounsaturated fatty acids (MUFA) and polyunsaturated fatty acids (PUFA) in the ovary (**a**), mantle muscle (**b**), and digestive gland (**c**) of female *D. gigas*. Data are presented as mean ± standard deviation.

SFA content was significantly lower and PUFA significantly higher in the ovaries of mature animals (stages IV and V) than in those of immature animals (stages II and III) (SFA *F* = 5.19, *P* = 0.008; PUFA *F* = 9.29, *P* = 0.0005; Fig. 1a). The higher PUFA content in the ovary of mature animals is expected given it is essential for egg and larval quality^35,44,45^. However, no individual fatty acid in the ovary differed significantly between animals at different maturity stages (Supplementary Tables 1 and 5). There were no significant differences in the proportion of the main fatty acid classes (SFA, MUFA and PUFA), nor in the relative amount of each fatty acid except 20:5n3, between maturity stages in the mantle muscle (Fig. 1b; Supplementary Tables 2 and 5). Similarly, the content of SFA and PUFA in the digestive gland was found vary, but not differ significantly, among maturity stages (SFA *F* = 1.05, *P* = 0.39; PUFA *χ*^2^ = 1.81, *P* = 0.61; Fig. 1c, Supplementary Table 5), and MUFA content and the relative amount of each fatty acid were also not significantly different among maturity stages (Supplementary Tables 3 and 5).

**Figure 2:**
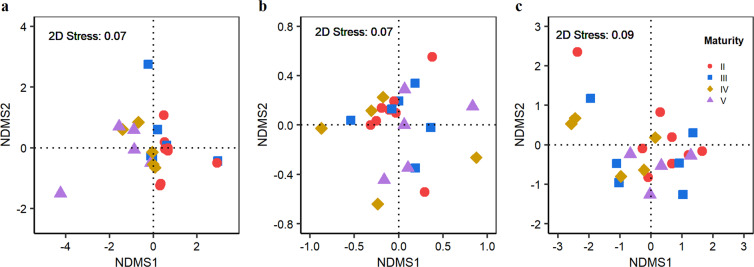
Non-metric Multidimensional Scaling (NMDS) ordination of fatty acid composition for each tissue by maturity stage. (**a**) ovary; (**b**) mantle muscle; and (**c**) digestive gland.

Multivariate analyses revealed considerable overlap in the overall fatty acids between maturity stages for each tissue (Fig. 2). A small but insignificant difference was detected for overall fatty acids between the ovaries of the four maturity stages (ANOSIM *R*-value = 0.11, *P* = 0.07), but no significant differences were found for the mantle muscle (ANOSIM *R*-value = 0.04, *P* = 0.28) and the digestive gland (ANOSIM *R*-value = 0.02, *P* = 0.36).

### Similarity of fatty acid composition between tissues

Paired Tests revealed that 11, 9 and 13 of the comparisons of the relative amount of each fatty acid between the ovary and the mantle muscle, between the ovary and the digestive gland, and between the mantle muscle and the digestive gland, respectively were significant for animals at maturity stage II (Supplementary Table 7). These numbers were 9, 7 and 12 for animals at maturity stage III (Supplementary Table 9), 6, 5 and 4 for animals at maturity stage IV (Supplementary Table 11), and 5, 4 and 7 for animals at maturity stage V (Supplementary Table 13).

**Figure 3:**
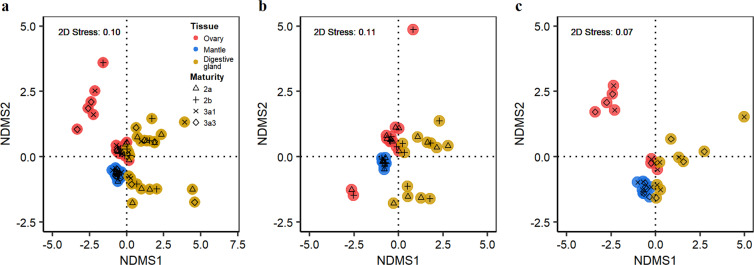
Non-metric Multidimensional Scaling (NMDS) ordination of fatty acid composition between the ovary, muscle tissue and digestive gland for female *D. gigas*. (**a**) pooled over maturity stages; (**b**) immature animals (stages II and III); (**c**) mature animals (stages IV and V).

Multivariate analyses revealed that the fatty acid profiles for the ovary overlapped those for the digestive gland (ANOSIM *R*-value = 0.32, *P* = 0.001), but not those for the mantle muscle (ANOSIM *R*-value = 0.54, *P* = 0.001), and that changes in the fatty acid composition for the ovary and the digestive gland showed a similar dispersed distribution pattern compared to a concentrated pattern for the mantle muscle (Fig. 3). There is greater similarity in fatty acid compositions between the ovary and the digestive gland during physiological maturation (stage III, *R*-value = 0.26, *P* = 0.012) and for physiologically mature animals (stage IV, *R*-value = 0.20, *P* = 0.026). This is also the case for the ovary and the mantle muscle, even though the extent of similarity was consistently less than that between the ovary and the digestive gland (Table 2). The extent of dissimilarity between the mantle muscle and the digestive gland was less for physiologically mature animals than animals at other maturity stages (Table 2).

**Table 2:** Results of analysis of similarity (ANOSIM) for the differences in fatty acid composition between the ovary, mantle muscle and digestive gland for female *D. gigas* by maturity stage.

ANOSIM pairwise tests							
Maturity stage	N	Ovary-Digestive gland		Ovary-Mantle		Mantle-Digestive gland	
		R value	Significance	R value	Significance	R value	Significance
II	8	0.46	0.002	0.74	0.001	0.59	0.001
III	6	0.26	0.012	0.53	0.002	0.54	0.002
IV	5	0.20	0.026	0.46	0.023	0.32	0.028
V	5	0.41	0.008	0.65	0.002	0.56	0.001
pooled	24	0.32	0.001	0.54	0.001	0.57	0.001

The R value of ANOSIM ranges from −1 to 1; values close to 0 indicates high similarity.

### Body condition, gonadosomatic index and feeding intensity

**Figure 4:**
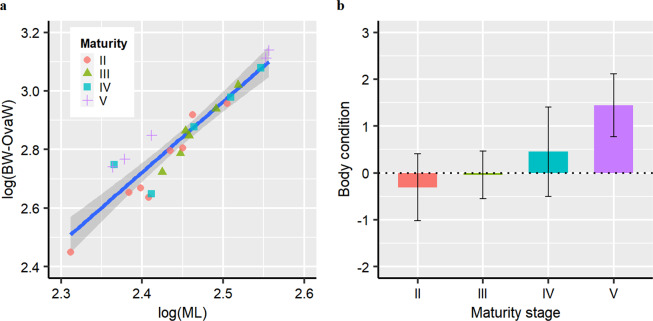
Linear regression between log mantle length and log body weight (**a**) and body condition distribution by maturity stage (**b**). Body condition is represented by the standardized residuals of a linear regression of log-body weight excluding ovary weight (log(BW-OvaW)) on log-mantle length (log(ML)). The solid blue line in (a) is the linear predictor (log(*BW-OvaW*) = −7.18 + 2.43 × log(*ML*); *r*^2^ = 0.90, *P* = 7.08e-13), with 95% confidence intervals in grey shading; Data in (b) are presented as mean ± standard deviation.

There was a significant positive correlation between body weight excluding ovary weight and mantle length (log(*BW-OvaW*) = −7.18 + 2.43 × log(*ML*); *r*^2^ = 0.90, *P* = 7.08e-13). Most functionally mature individuals (stage V) were heavier for a given length (Fig. 4a). Consequently, significant differences in body condition (represented by the residuals of body weight excluding ovary weight regressed on the mantle length) were found between maturity stages (ANOVA, *F* = 6.67, *P* = 0.003; Fig. 4b).

**Figure 5:**
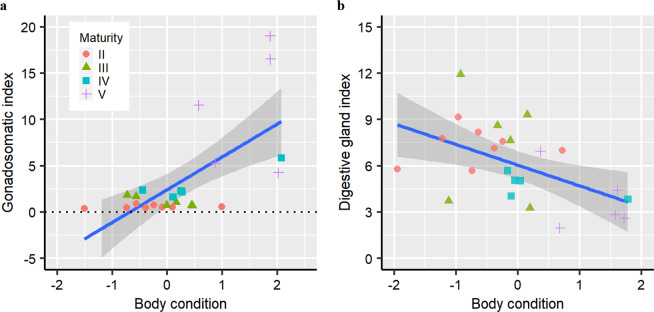
Relationship between body condition and gonadosomatic index (**a**) and digestive gland index (**b**). Body condition (BC) is represented by the standardized residuals from the linear regression of log body weight excluding ovary weight on log mantle length. The solid blue lines are from linear regressions (a, *GSI* = 2.43 + 3.54 × *BC*; *r*^2^ = 0.41, *P* = 0.0004. b, *DGI* = 6.41–1.29 × *BC*; *r*^2^ = 0.21, *P* = 0.014), with 95% confidence intervals in grey shading.

The gonadosomatic index (GSI) increased significantly following maturation (K-W test, *χ*^2^ = 19.05, *P* = 0.0002; II: 0.58 ± 0.17 (range 0.39–0.92); III: 1.12 ± 0.52 (range 0.67–1.81); IV: 2.86 ± 1.69 (range 1.63–5.84); V: 11.31 ± 6.57 (range 4.25–19.00)). There was a weak but significant correlation between GSI and body condition (GSI = 2.43 + 3.54 × BC; *r*^2^ = 0.41, *P* = 0.0004; Fig. 5a), suggesting that reproductive allocation is higher when animals are heavier than expected given their lengths.

In contrast, the digestive gland index (DGI) was lower following maturation (ANOVA, *F* = 4.64*, P* = 0.012; II: 7.28 ± 1.17 (range 5.68–9.16); III: 7.41 ± 3.36 (range 3.25–11.93); IV: 4.72 ± 0.78 (range 3.82–5.68); V: 3.75 ± 2.00 (range 1.96–6.94)). DGI was also negatively related to body condition (DGI = 6.41–1.29 × BC; *r*^2^ = 0.21, *P* = 0.014; Fig. 5b). These observations indicate that individuals reduce feeding prior to reproduction, but maintain body condition.

## Discussion

Data on fatty acids show that *D. gigas* preys on similar organisms during ontogeny, given the same pattern of fatty acid profiles for animals at different maturity stages in all tissues. Energy for reproduction appears to be driven primarily by concurrent food intake, since the changes in fatty acids in the ovary closely resemble those in the digestive gland. The fatty acid compositions of the ovary and the mantle muscle change similarity with maturation, most notably from the developing to physiologically mature stages, indicating that energy reserves are also involved in reproduction. Female *D. gigas* appear to maintain somatic fitness, although the investment in reproduction increases with maturation along with a major reduction in feeding intensity. As such, female *D. gigas* adopt a mixed income-capital breeding strategy, which mostly relies on continual food intake, coupled with the limited use of stored energy during sexual maturation.

Of the tissues analysed in female *D. gigas*, the digestive gland has the greatest fatty acid content regardless of maturity stage (Table 1, Supplementary Tables 1–3). However, the predominant fatty acids in the main fatty acid classes (SFA, MUFA, PUFA) are similar for each tissue, with 16:0 and 18:0 most prevalent in SFA, 18:1n9c and 20:1 most prevalent in MUFA, and 20:5n3 and 22:6n3 most prevalent in PUFA (Table 1). The most prevalent fatty acids may be the result of the fatty acid levels of the diet sources given the limited capacity for biosysnthesis of fatty acids^33,34,36^. On the other hand, these oberservations are in accordance with the findings of Saito *et al*.^46^ and Gong *et al*.^47^, and are also very similar to the results of studies for other squids such as *Loligo vulgaris*^48^ and *Todarodes filippovae*^49^. This may imply that these fatty acids are the common nutrients for squids, presumably owing to their important roles in cell and organelle function^31,50,51^, as well as energy sources for rapid growth and development^41,52,53^. The significantly lower SFA content in the ovaries of mature animals could be due to energy mobilization for reproductive growth^53,54^.

Studies on trophic relationships have shown that many species, including cephalopods change feeding habits with increasing size or during maturation to maximize energy intake, enhance growth rate and minimize the risk of predation^55–60^. In the present study, however, the female *D. gigas* appear to prey on similar prey items before and after maturation because no significant differences were found in the relative abundance of each fatty acid among maturity stages for the ovary, mantle muscle (except 20:5n3) and digestive gland (Supplementary Table 5). Further evidence is provided by the clear overlap and similarity of the overall fatty acid profiles between maturity stages for each tissue (Fig. 2). These findings indicate that the female *D. gigas* may adopt a foraging strategy that focuses on the amount and not quality of food, which is not unexpected, as squids are well known for their voracious and opportunistic feeding^3,12^. Although squids seem to become more active and successful predators as they mature^3^, their energy expenditure is higher given the need for increased metabolism with maturation for basic maintenance, predation and reproductive growth^61,62^. Preying on species that are caught more easily may be a successful tactic to balance energy expenditure during the period of reproduction when energy requirements are relatively high^6,7,26,42^. Indeed, studies have showed that squids including *D. gigas* are opportunitistic predators at all maturity stages^12,63,64^, presumably related to their “live for today” lifestyle^10^.

Among the tissues analysed, consistently fewer fatty acids differed significantly between the ovary and the digestive gland than between the ovary and the mantle muscle for any maturity stage (Supplementary Tables 7, 9, 11 and 13), and this was supported by the multivariate analyses (Fig. 3, Table 2). These lines of evidence indicate that there is an energy trade-off between gonad development and resource uptake, with the energy sources for reproduction derived primarily from concurrent intake of prey. The similar despersed distribution pattern of the fatty acid composition for the ovary and the digestive gland revealed by the NMDS analyses (Fig. 3) might indirectly provide futher evidence of energy allocation to reproduction acquired directly from food intake, as the fatty acids in the digestive gland reflect the corresponding diets within a more recent period of 10–14 days^39,40^. Furthermore, the fatty acid composition between the ovary and the digestive gland is more similar for mature animals (particularly those that are physiologically mature) (Table 2), suggesting an increase in energy allocation to reproduction from food intake, which is consistent with the gonadosomatic index (GSI) being significantly higher for mature animals (K-W test, *χ*^2^ = 19.05, *P* = 0.0002). It is worth noting that the fatty acid composition in the ovary and the digestive gland appears to vary at the individual level, especially for the ovary at the functionally mature stage (Fig. 3). A possible reason for this is the fact that squids prey on a wide spectrum of prey items^12,63,64^. Variation in the fatty acids of the ovary at the mature stage may be also related to the accumulation of essential fatty acids such as long-chain polyunsaturated fatty acids for egg quality^35,44,45^ and possible remobilization of short-chain saturated fatty acids for energy use^53,54^, since the ovary showed a significant increase of PUFA content and decrease of SFA content with maturation (Fig. 1). However, future studies on the specific fatty acid requirements for gonad development are needed to address these hypotheses.

Reproduction generally constitutes a major fraction of the total energy budget of an adult organism^1^, and gonad development in many organisms is fuelled by increased food intake as well as mobilization of previously stored reserves^65^. In the present study, although the fatty acid composition in the ovary differed from that in the mantle muscle (Fig. 3), the similarity in fatty acid composition between these two tissues increased from the developing to physiologically mature stage (Table 2). Meanwhile, the significant reduction of the digestive gland index (ANOVA, *F* = 4.64*, P* = 0.012), an index of feeding activity^38,66^, for mature females suggested a possible reduction in feeding intensity during maturation. It is therefore reasonable to expect that mature female *D. gigas* remobilize some of their somatic reserve to provide energy for reproduction.

The energy remobilization of somatic reserves for reproduction is limited and probably only occurs as a complementary source during maturation when the development of the reproductive organs is significant. This is because the dissimilarity (represented by the ANOSIM *R*-value) in the fatty acid composition between the ovary and the mantle muscle is larger than that between the ovary and the digestive gland (*R*-value, 0.54 *vs*. 0.32). Meanwhile, female *D. gigas* have better body condition when mature (Fig. 4B), indicating that the adults have not used up much somatic tissue. Further, the animals with higher reproductive investment appeared to be in good condition (Fig. 5A), although they fed less given the negative relationship between body condition and the digestive gland index (Fig. 5B). These lines of evidence suggest that female *D. gigas* maintain somatic fitness even if some energy reserves are mobilized. Indeed, the fatty acid composition in the mantle muscle more closely resembles that of the digestive gland for mature animals (Table 2), suggesting that the somatic tissues continue to incorporate nutrients from feeding. This is in stark contrast to species with synchronous ovarian development, such as *O. ingens* that mobilizes much of its somatic tissues to support reproduction^6^. The pattern of limited use of somatic reserve for reproduction may be an evolutionary tactic to adapt to the asynchronous ovarian development of *D. gigas*^2,19^, as the maintenance of somatic condition appears to be important for this species to develop the multiple cohorts of oocytes during the protracted spawning period^20,21^.

## Conclusions

Female *D. gigas* feed on similar prey items during ontogeny, and adopt a mixed income-capital breeding strategy, in which energy for reproduction is mainly derived from direct feeding, coupled with limited mobilization of somatic energy. The results confirm the recent suggestion by Han *et al*.^26^ that the energy reserves in the somatic tissues are remobilized to support reproduction during maturation. The energy trade-off between reproduction and limited use of energy reserve warrants further research to better understand the life-history strategy of *D. gigas* in terms of energy acquisition and allocation both within and across taxa. This study could also contribute to the use of fatty acids as biochemical markers to identify the breeding strategy for oceanic squids as suggested by Lin *et al*.^42^.

## Methods

### Ethics statement

Specimens were collected as dead squids from the commercial jigging fisheries landings, during the fishing season from June to August 2017. The specimens were analyzed in the laboratory using methods that are in line with current Chinese national standards, namely Laboratory Animals - General Requirements for Animal Experiment (GB/T 35823-2018). As all material sampled in this work was obtained from commercial fishermen and already dead, there was no requirement for ethical approval of sampling protocols as it did not include live organisms.

### Sample collection

Samples were collected from the landings from commercial jig fishery in the eastern Pacific (longitude: 84°07′W~102°27′W, latitude: 00°47′S~08°26′S), from June to August 2017. These were immediately frozen at −30 °C for further analyses in the laboratory.

A total of 24 females (14 immature and 10 mature) were randomly selected for the following fatty acids analyses after defrosting at room temperature in the laboratory. Each specimen was assigned a maturity stage following the scheme proposed by Arkhipkin^67^, Arkhipkin and Laptikhovsky^68^ and ICES^69^, with maturity stages: I immature, II developing, III physiologically maturing, IV physiologically mature, V functionally mature, VI spawning, and VII spent. Specimens were in maturity stages II to V (Table 3). Specimens at maturity stages II and III were categorized as immature, and specimens at maturity stages IV and V as mature. The following parameters were also recorded for each specimen: mantle length (ML, mm), body weight (BW, g), ovary weight (OvaW, g) and digestive gland weight (DgW, g) (Table 3). The gonadosomatic index (GSI; ovary weight/body weight × 100) and the digestive gland index (DGI; digestive gland weight/body weight × 100) were also determined for each specimen^38,70^.

**Table 3:** Summary of biological measurements for female *Dosidicus gigas* collected from landings from commercial jig fishery in the eastern Pacific.

Maturity stage	n	Mantle length (ML, mm)	Body weight (BW, g)	Ovary weight (OvaW, g)	Digestive gland weight (DgW, g)
II	8	264.6 ± 34.7	578.5 ± 211.7	3.3 ± 1.3	41.6 ± 14.8
III	6	282.8 ± 23.1	749.0 ± 186.6	7.9 ± 2.4	54.7 ± 20.5
IV	5	291.2 ± 48.3	782.2 ± 303.0	20.4 ± 8.8	38.4 ± 18.9
V	5	289.9 ± 64.2	902.4 ± 401.0	86.9 ± 47.9	34.6 ± 22.0
Pooled	24	283.4 ± 42.7	743.5 ± 303.5	26.1 ± 39.4	43.8 ± 21.1

Data are presented as mean ± standard deviation.

The ventral mantle muscle (~10.0 g), whole gonad and whole digestive gland were collected for each individual, and separately lyophilized to a constant weight in a freeze-drying system (Christ Alpha 1–4/LDplus). The digestive gland is a site of digestive absorption and intracellular digestion^37,38^, and deposits recent intake of dietary fatty acids (10–14 days) without modification^36,39,40,71^. The mantle muscle is the most important energy reserve organ^7,42,72^, and reflects dietary information over a time scale of 4 weeks or longer^39^.The dried tissues were ground to fine powder individually, and a 0.2 g subsample was used for fatty acid analysis.

### Fatty acid analyses

Fatty acid methyl esters (FAME) were analyzed for each tissue sample of each specimen following the “Determination of total fat, saturated fat, and unsaturated fat in foods - Hydrolytic extraction-gas chromatography” protocol^73^. Lipids were extracted by using a mixture of chloroform and methanol 2:1 (v/v)^74^. To esterify the fatty acids, lipids were introduced into a 25 mL vial with 4 mL of 0.5 mol/L KOH-MeOH, which was incubated at 90 °C for 10 minutes, shaking for 5 seconds every 2 minutes. Then, 4 ml BF3/MeOH was added and the sample incubated at 90 °C for 30 minutes, shaking for 5 seconds every 5 minutes, followed by the addition of 4 mL n-Hexane for 2 minutes incubation at a similar temperature. Thirdly, 10 mL saturated NaCl was added and shaken gently, followed by introduction into a 20 mL centrifuge tube for stratification at room temperature. Finally, the upper hexane layer, which contained the FAME, was transferred to a vial, and evaporated under nitrogen current with 19:0 as an internal standard.

Fatty acids were determined using an Agilent 7890B Gas Chromatography (GC) coupled to a 5977 A series Mass Spectrometer Detector (MSD, Agilent Technologies, Inc. USA), equipped with a fused silica 60 m × 0.25 nm open tubular column (HB-88: 0.20 μm, Agilent Technologies, Inc. USA). The separation was carried out with helium as the carrier gas, and a thermal gradient programed from 125 °C to 250 °C, with the auxiliary heater at 280 °C. Individual fatty acid peaks were identified by comparing their retention times with those of chromatographic Sigma standards. Total fatty acids (total FAs) were determined as mg/g, and individual fatty acids were expressed as percentages of total fatty acids (% of total fatty acids)^36^. The individual fatty acids were also grouped into saturated fatty acids (SFA), monounsaturated fatty acids (MUFA), and polyunsaturated fatty acids (PUFA). Fatty acids that accounted for <0.5% were excluded from statistical analyses.

### Statistical analysis

The results were expressed as means ±standard deviation. The fatty acid data for each tissue were checked for normality using the one-sample Kolmogorov-Smirnov test^75^ (Supplementary Tables 4, 6, 8, 10 and 12). Thereafter, one-way analysis of variance (ANOVA) was used to detect significant differences in the means of the main fatty acid classes (SFA, MUFA, PUFA) and each fatty acid between maturity stages for each tissue^75^. When normality was rejected, the data were analyzed using Kruskal-Wallis tests (K-W test)^75^. Paired *t*-Tests were used to investigate significant differences for each fatty acid within matched pairs of tissues given maturity stage, and paired Wilcoxon tests were used when normality was rejected^75^.

Non-metric multidimensional scaling (NMDS) and analysis of similarities (ANOSIM) were applied to assess the differences in the overall fatty acid profiles between immature and mature stages for each tissue, and to determine the differences in the overall fatty acids between the ovary, the mantle muscle and the digestive gland. These multivariate analyses of fatty acids have the advantage of pattern recognition^28,76^, and can be used to determine whether energy for reproduction is from energy reserves (mantle tissue) or consumption of prey (digestive gland)^42^. The fatty acid data were square root transformed and Euclidean dissimilarity matrices were used in the NMDS and ANOSIM^77^. NMDS and ANOSIM analyses were conducted using the vegan package in R^78^.

The relationship between mantle length (ML) and body weight (BW) excluding ovary weight (OvaW) was examined after log-transformation, and the standardized residuals of the regression used as an index of body condition^6,22^, where the residuals provide a size-independent measure of the somatic condition of an individual at the whole animal level^5,6^. ANOVA was used to detect differences in the means of body condition, GSI and DGI between maturity stages, and these data were analyzed using Kruskal-Wallis tests when the normality assumption was not satisfied^75^.

To some extent, the GSI can be used as an indicator of reproductive investment^5^, while the DGI can be used as an indicator of feeding intensity^38^. The linear relationships among body condition, GIS and DGI were investigated to assess the interactions between the soma reserve, reproduction and energy acquisition.

Statistical analyses were carried out using SPSS 20.0 and R version 3.5.0^78^. A test was considered significant when *P* < 0.05.

## Supplementary information

Supplementary Information.

## Data Availability

The biological measurement data and biochemical data (fatty acids) that support the findings of this study are available from the Distant Squid Fisheries Sci-Tech Group (SHOU), but restrictions apply to the availability of these data, which were used under license for the current study, and so are not publicly available. Data are however available from the authors upon reasonable request and with permission of the Distant Squid Fisheries Sci-Tech Group (SHOU).
