# Supplementary material for: The breeding strategy of female jumbo squid Dosidicus gigas: energy acquisition and allocation
Source: Sci Rep. 2020 Jun 15;10:9639. doi: 10.1038/s41598-020-66703-5 (PMC7295804; doi:10.1038/s41598-020-66703-5)
Supplement: Supplementary file 1 — Supplementary Information. [file 41598_2020_66703_MOESM1_ESM.pdf]

The breeding strategy of female jumbo squid *Dosidicus gigas*: energy acquisition and allocation

Xinjun Chen, Fei Han, Kai Zhu, André E. Punt, Dongming Lin

Supplementary Table 1 Fatty acid composition in the ovary of female *Dosidicus gigas*.

Fatty acid	Maturity stage			
	II	III	IV	V
Fatty acid (% total FAs*)*				
14:0	1.09±0.51	1.71±1.75	1.73±1.17	3.62±2.78
15:0	0.42±0.20	0.43±0.31	0.45±0.17	0.62±0.48
16:0	19.51±3.81	16.30±8.09	10.53±9.65	3.27±7.31
17:0	1.26±0.27	1.70±1.00	1.63±0.64	2.98±2.62
18:0	7.50±3.12	10.42±7.61	10.89±3.62	8.58±5.89
20:0	0.39±0.30	0.27±0.05	0.28±0.07	0.27±0.18
SFA	32.09±6.03	32.00±5.25	26.95±5.75	20.72±5.16
16:1n7	0.49±0.23	0.49±0.19	0.47±0.13	0.50±0.20
17:1n7	1.22±3.12	1.65±3.95	0.05±0.07	0.01±0.03
18:1n9c	2.65±1.32	4.05±4.23	4.14±2.27	5.07±4.46
20:1	12.03±1.74	13.45±2.30	13.43±1.13	10.13±4.88
22:1n9	0.38±0.15	0.32±0.05	0.35±0.06	0.37±0.21
24:1n9	0.34±0.13	0.29±0.06	0.34±0.05	0.34±0.18
MUFA	18.12±3.03	21.02±6.70	19.26±2.70	16.92±2.76
18:2n6c	0.34±0.19	0.37±0.17	0.29±0.16	0.24±0.14
20:2	0.80±0.25	0.96±0.46	0.83±0.30	0.79±0.36
20:3n3	3.97±0.95	3.82±0.88	3.89±2.05	2.78±1.78
20:4n6(ARA)	5.42±1.55	5.37±1.30	5.81±2.01	5.08±1.70
22:2n6	1.23±2.04	0.11±0.02	0.16±0.06	0.09±0.04
20:5n3(EPA)	15.16±2.13	13.13±4.16	15.99±3.84	23.63±16.85
22:6n3(DHA)	22.40±1.68	22.88±2.66	26.44±7.95	28.51±17.61
PUFA	49.79±4.32	46.98±7.00	53.79±4.56	62.36±4.18
∑FAs<0.5%	5.14±3.43	3.86±0.98	4.26±0.57	5.11±2.70
Total fatty acids (mg/g dry weight)				
total FAs	69.53±10.22	91.42±3.95	124.98±22.93	169.86±63.19

FAs <0.5% include 15:0, 20:0, 16:1n7, 17:1n7, 22:1n9, 24:1n9, 18:2n6c, 20:2, 22:2n6. ARA, arachidonic acid; EPA, eicosapentaenoic acid; DHA, docosahexaenoic acid; SFA, saturated fatty acids; MUFA, monounsaturated fatty acids; PUFA, polyunsaturated fatty acids; total FAs, total fatty acids. Values are mean ± standard deviation; total FAs is reported as dry tissue weight (mg/g dry weight), other values are reported as percentages of total FAs (%total FAs).

Supplementary Table 2 Fatty acid composition in the mantle muscle of female *Dosidicus gigas*.

Fatty acid	Maturity stage			
	II	III	IV	V
Fatty acid (% total FAs*)*				
14:0	0.74±0.11	0.82±0.08	0.73±0.05	0.77±0.10
15:0	0.47±0.06	0.51±0.11	0.44±0.05	0.54±0.11
16:0	23.12±1.51	23.08±1.97	20.46±2.42	23.34±1.92
17:0	1.00±0.14	0.98±0.14	0.97±0.11	1.05±0.13
18:0	5.87±0.32	5.77±0.64	6.21±0.68	5.59±0.39
20:0	0.24±0.03	0.25±0.03	0.21±0.02	0.29±0.11
SFA	33.09±1.85	33.14±1.82	30.43±1.91	33.67±2.65
16:1n7	0.33±0.05	0.35±0.06	0.31±0.04	0.37±0.07
17:1n7	0.00±0.00	0.05±0.07	0.03±0.05	0.05±0.08
18:1n9c	1.53±0.13	1.54±0.26	1.48±0.19	1.67±0.39
20:1	6.07±1.06	5.89±0.50	6.95±1.21	5.64±1.33
22:1n9	0.21±0.02	0.22±0.03	0.20±0.02	0.26±0.06
24:1n9	0.17±0.01	0.18±0.02	0.15±0.02	0.21±0.07
MUFA	8.73±0.98	8.63±0.77	9.50±1.31	8.76±1.55
18:2n6c	0.17±0.03	0.15±0.08	0.15±0.02	0.18±0.11
20:2	0.33±0.04	0.40±0.13	0.33±0.05	0.41±0.09
20:3n3	0.76±0.56	0.91±0.61	0.77±0.66	1.37±0.58
20:4n6(ARA)	2.09±0.38	2.02±0.30	1.79±0.27	2.32±0.53
22:2n6	0.12±0.08	0.11±0.09	0.15±0.02	0.22±0.10
20:5n3(EPA)	11.89±1.04	11.72±0.86	13.43±0.90	11.07±1.58
22:6n3(DHA)	42.40±1.24	42.42±1.31	42.99±1.39	41.43±1.83
PUFA	58.18±1.21	58.23±1.37	60.07±1.51	57.57±1.88
∑FAs<0.5%	4.05±0.56	4.33±0.50	3.77±0.33	5.20±1.76
Total fatty acids (mg/g dry weight)				
total FAs	65.28±15.04	74.12±11.43	112.19±10.14	151.63±30.17

FAs <0.5% include 15:0, 20:0, 16:1n7, 17:1n7, 22:1n9, 24:1n9, 18:2n6c, 20:2, 22:2n6. ARA, arachidonic acid; EPA, eicosapentaenoic acid; DHA, docosahexaenoic acid; SFA, saturated fatty acids; MUFA, monounsaturated fatty acids; PUFA, polyunsaturated fatty acids; total FAs, total fatty acids. Values are mean ± standard deviation; total FAs is reported as dry tissue weight (mg/g dry weight), other values are reported as percentages of total FAs (%total FAs).

Supplementary Table 3 Fatty acid composition in the digestive gland of female *Dosidicus gigas*.

Fatty acid	Maturity stage			
	II	III	IV	V
Fatty acid (% total FAs*)*				
14:0	5.16±1.47	3.85±1.62	3.98±1.17	4.68±1.15
15:0	1.14±0.53	0.95±0.45	0.87±0.64	1.06±0.47
16:0	27.18±4.48	23.72±7.99	21.12±3.96	24.75±9.33
17:0	1.80±0.84	1.70±0.71	1.30±0.94	1.92±0.68
18:0	9.50±2.38	8.96±1.29	7.90±2.15	9.67±2.15
20:0	0.54±0.15	0.49±0.15	0.44±0.22	0.52±0.21
SFA	46.58±8.82	40.79±11.37	36.50±8.36	43.78±12.94
16:1n7	4.54±2.38	3.51±2.21	3.43±1.42	3.41±1.71
17:1n7	1.28±0.53	1.03±0.49	0.83±0.30	1.32±0.51
18:1n9c	6.83±7.34	9.20±9.27	6.73±6.24	8.67±8.17
20:1	5.36±2.37	6.54±1.74	7.91±3.55	4.84±3.27
22:1n9	0.41±0.10	0.43±0.08	0.48±0.21	0.74±0.59
24:1n9	1.06±0.42	1.05±0.13	1.13±0.31	1.34±0.49
MUFA	19.83±7.36	22.13±8.50	20.83±2.93	20.67±5.91
18:2n6c	1.17±0.36	0.93±0.41	0.81±0.23	1.05±0.30
20:2	1.19±0.25	1.31±0.59	1.58±1.11	1.38±0.49
20:3n3	0.83±0.89	0.77±0.71	1.29±0.76	0.92±0.63
20:4n6(ARA)	3.92±1.97	3.62±1.05	4.37±0.69	3.82±1.83
22:2n6	0.13±0.05	0.14±0.04	0.14±0.06	0.15±0.02
20:5n3(EPA)	8.47±4.43	7.15±2.70	10.57±1.87	9.11±5.00
22:6n3(DHA)	16.84±10.52	22.30±10.37	22.89±13.24	18.20±11.97
PUFA	33.59±12.22	37.08±13.20	42.67±10.54	35.56±10.83
∑FAs<0.5%	2.77±0.57	2.47±0.34	2.36±0.61	2.59±0.40
Total fatty acids (mg/g dry weight)				
total FAs	165.70±51.94	171.44±80.25	288.18±73.36	368.10±108.48

FAs <0.5% include 15:0, 20:0, 16:1n7, 17:1n7, 22:1n9, 24:1n9, 18:2n6c, 20:2, 22:2n6. ARA, arachidonic acid; EPA, eicosapentaenoic acid; DHA, docosahexaenoic acid; SFA, saturated fatty acids; MUFA, monounsaturated fatty acids; PUFA, polyunsaturated fatty acids; total FAs, total fatty acids. Values are mean ± standard deviation; total FAs is reported as dry tissue weight (mg/g dry weight), other values are reported as percentages of total FAs (%total FAs).

Supplementary Table 4. The results of the one-sample Kolmogorov-Smirnov test for each fatty acid within each tissue analysed for *Dosidicus gigas*.

Fatty acid	Ovary		Mantle muscle		Digestive gland	
	statistic	p.value	statistic	p.value	statistic	p.value
14:0	0.71	**0.00**	0.98	0.82	0.97	0.75
15:0	0.71	**0.00**	0.92	**0.07**	0.86	**0.00**
16:0	0.83	**0.01**	0.96	0.45	0.97	0.57
17:0	0.58	**0.00**	0.95	0.30	0.95	0.24
18:0	0.88	**0.01**	0.97	0.56	0.94	0.21
20:0	0.68	**0.00**	0.76	**0.00**	0.94	0.14
16:1n7	0.87	**0.00**	0.94	0.21	0.93	0.08
17:1n7	0.58	**0.00**	0.94	0.64	0.87	**0.01**
18:1n9c	0.81	**0.00**	0.83	**0.00**	0.90	0.06
20:1	0.77	**0.00**	0.98	0.89	0.95	0.21
22:1n9	0.82	**0.00**	0.81	**0.00**	0.69	**0.00**
24:1n9	0.86	**0.00**	0.70	**0.00**	0.93	0.09
18:2n6c	0.90	**0.02**	0.78	**0.00**	0.96	0.41
20:2	0.96	0.47	0.81	**0.00**	0.78	**0.00**
20:3n3	0.85	**0.00**	0.77	**0.00**	0.87	**0.01**
20:4n6	0.89	**0.01**	0.92	0.06	0.96	0.48
22:2n6	0.35	**0.00**	0.74	**0.00**	0.94	0.15
20:5n3	0.58	**0.00**	0.97	0.60	0.94	0.15
22:6n3	0.75	**0.00**	0.96	0.46	0.96	0.41
SFA	0.97	0.62	0.97	0.69	0.92	0.05
MUFA	0.80	**0.00**	0.97	0.68	0.96	0.38
PUFA	0.94	0.15	0.98	0.93	0.90	**0.02**
TFA	0.82	**0.00**	0.69	**0.00**	0.93	0.11

SFA, saturated fatty acids; MUFA, monounsaturated fatty acids; PUFA, polyunsaturated fatty acids; TFA, total fatty acids. P.value highlighted in bold indicates significant differences (P<0.05).

Supplementary Table 5. Results of tests for significant differences in fatty acids between maturity stages for the ovary, the mantle muscle and the digestive gland of *Dosidicus gigas*.

**Ovary**			**Digestive gland**			**Mantle muscle**		
Fatty acid	statistic	p.value	Fatty acid	statistic	p.value	Fatty acid	statistic	p.value
ANOVA comparing the FAs satisfying normality			ANOVA comparing the FAs satisfying normality			ANOVA comparing the FAs satisfying normality		
20:2	0.33	0.80	14:0	1.29	0.31	14:0	1.12	0.36
SFA*	5.19	0.01	16:0	0.91	0.45	15:0	1.39	0.28
PUFA*	9.29	0.00	17:0	0.59	0.63	16:0	2.32	0.11
Kruskal-Wallis tests comparing the FAs not satisfying normality			18.0	0.80	0:51	17.0	0.33	0:80
14:0	3.16	0.37	20:0	0.36	0.78	18:0	1.28	0.31
15:0	2.04	0.56	16:1n7	0.50	0.69	16:1n7	1.40	0.27
16:0*	10.31	0.02	18:1n9c	0.16	0.92	17:1n7	1.10	0.37
17:0	4.34	0.23	20:1	1.35	0.29	20:1	1.32	0.30
18:0	2.16	0.54	24:1n9	0.75	0.53	20:4n6	1.56	0.23
20:0	2.50	0.48	18:2n6c	1.23	0.32	20:5n3*	3.51	0.03
16:1n7	0.32	0.96	20:4n6	0.22	0.88	22:6n3	1.04	0.40
17:1n7	1.51	0.68	22:2n6	0.16	0.92	SFA	2.18	0.12
18:1n9c	1.01	0.80	20:5n3	0.77	0.52	MUFA	0.53	0.66
20:1	4.57	0.21	22:6n3	0.43	0.73	PUFA	2.38	0.10
22:1n9	0.95	0.81	SFA	1.05	0.39	Kruskal-Wallis tests comparing the FAs not satisfying normality		
24:1n9	1.49	0.68	MUFA	0.13	0.94	20:0	4.58	0.21
18:2n6c	1.71	0.64	TFA*	9.21	0.00	18:1n9c	1.13	0.77
20:3n3	2.84	0.42	Kruskal-Wallis tests comparing the FAs not satisfying normality			22.1n9	4.62	0:20
20:4n6	1.40	0.71	15:0	2.71	0.44	24:1n9	4.79	0.19
22:2n6	6.46	0.09	17:1n7	4.63	0.20	18:2n6c	1.42	0.70
20:5n3	3.88	0.28	22:1n9	0.47	0.93	20:2	3.33	0.34
22:6n3	1.42	0.70	20:2	0.59	0.90	20:3n3	6.34	0.10
MUFA	1.83	0.61	20:3n3	2.61	0.46	22:2n6	4.30	0.23
TFA*	20.50	0.00	PUFA	1.81	0.61	TFA*	17.64	0.00

SFA, saturated fatty acids; MUFA, monounsaturated fatty acids; PUFA, polyunsaturated fatty acids; TFA, total fatty acids. Asterisk "*" indicates significant differences (P<0.05).

Supplementary Table 6. Results of one-sample Kolmogorov-Smirnov tests for each fatty acid content between paired tissues for *Dosidicus gigas* at maturity stage II.

Fatty acid	Ovary vs. Mantle		Ovary vs. Digestive gland		Mantle muscle vs. digestive gland	
	statistic	p.value	statistic	p.value	statistic	p.value
14:0	0.65	**0.00**	0.86	**0.02**	0.82	**0.01**
15:0	0.89	0.05	0.87	**0.02**	0.79	**0.00**
16:0	0.95	0.43	0.88	**0.03**	0.94	0.38
17:0	0.91	0.12	0.87	**0.02**	0.80	**0.00**
18:0	0.87	**0.03**	0.89	0.05	0.80	**0.00**
20:0	0.59	**0.00**	0.91	0.12	0.83	**0.01**
16:1n7	0.69	**0.00**	0.80	**0.00**	0.78	**0.00**
17:1n7	0.31	**0.00**	0.56	**0.00**	0.81	**0.00**
18:1n9c	0.91	0.14	0.77	**0.00**	0.67	**0.00**
20:1	0.91	0.10	0.94	0.37	0.77	**0.00**
22:1n9	0.78	**0.00**	0.95	0.54	0.86	**0.02**
24:1n9	0.76	**0.00**	0.88	**0.04**	0.81	**0.00**
18:2n6c	0.75	**0.00**	0.93	0.25	0.84	**0.01**
20:2	0.83	**0.01**	0.98	0.96	0.84	**0.01**
20:3n3	0.78	**0.00**	0.84	**0.01**	0.73	**0.00**
20:4n6	0.79	**0.00**	0.90	0.07	0.78	**0.00**
22:2n6	0.44	**0.00**	0.43	**0.00**	0.94	0.34
20:5n3	0.93	0.24	0.90	0.08	0.87	**0.03**
22:6n3	0.76	**0.00**	0.88	0.05	0.84	**0.01**

P.value highlighted in bold indicates significant differences (P<0.05).

Supplementary Table 7. Results of tests for significant differences in fatty acids between paired tissues for *Dosidicus gigas* at maturity stage II.

Ovary vs. Mantle			Ovary vs. Digestive gland			Mantle vs. Digestive gland		
Fatty acid	statistic	p.value	Fatty acid	statistic	p.value	Fatty acid	statistic	p.value
Paired *t*-Tests comparing the FAs satisfying normality			Paired *t*-Tests comparing the FAs satisfying normality			Paired *t*-Tests comparing the FAs satisfying normality		
15:0	0.73	0.49	20:0	1.33	0.23	16:0	2.33	0.05
16:0*	2.17	0.04	20:1*	-5.19	0.00	22:2n6	0.35	0.74
17:0*	-2.76	0.03	22:1n9	0.57	0.59	Paired Wilcoxon tests comparing the FAs not satisfying normality		
18:1n9c*	-2.26	0.04	18:2n6c*	5.04	0.00	14:0*	36.00	0.01
20:1*	-9.11	0.00	20:2	2.92	0.05	15:0*	36.00	0.01
20:5n3*	-4.91	0.00	20:4n6	-1.86	0.11	17:0*	33.00	0.04
Paired Wilcoxon tests comparing the FAs not satisfying normality			20.5n3*	-5.55	0:00	18.0*	36.00	0:01
14:0	6.00	0.11	Paired Wilcoxon tests comparing the FAs not meet normality			20.0*	36.00	0:01
18:0	8.00	0.20	14:0*	36.00	0.01	16:1n7*	36.00	0.01
20:0	10.00	0.31	15:0*	36.00	0.01	17:1n7*	36.00	0.01
16:1n7	5.00	0.08	16:0*	36.00	0.01	18:1n9c	26.00	0.31
17:1n7	0.00	0.18	17:0	31.00	0.08	20:1	16.00	0.84
22:1n9*	0.00	0.01	18:0	31.00	0.08	22:1n9*	36.00	0.01
24:1n9*	0.00	0.01	16:1n7*	36.00	0.01	24:1n9*	36.00	0.01
18:2n6c	5.00	0.08	17:1n7	28.00	0.20	18:2n6c*	36.00	0.01
20:2*	1.00	0.02	18:1n9c	26.00	0.31	20:2*	36.00	0.01
20:3n3*	0.00	0.01	24:1n9*	36.00	0.01	20:3n3	19.00	0.95
20:4n6*	1.00	0.02	20:3n3*	0.00	0.01	20:4n6*	33.00	0.04
22:2n6	13.00	0.55	22:2n6	9.00	0.25	20:5n3	4.00	0.05
22:6n3*	36.00	0.01	22:6n3	7.00	0.15	22:6n3*	0.00	0.01

Asterisk “*” indicates significant differences (P<0.05).

Supplementary Table 8. Results of one-sample Kolmogorov-Smirnov tests for each fatty acid content between paired tissues for *Dosidicus gigas* at maturity stage III.

Fatty acid	Ovary vs. Mantle		Ovary vs. Digestive gland		Mantle vs. digestive gland	
	statistic	p.value	statistic	p.value	statistic	p.value
14:0	0.45	**0.00**	0.90	0.15	0.82	**0.02**
15:0	0.83	**0.02**	0.84	**0.03**	0.74	**0.00**
16:0	0.67	**0.00**	0.90	0.18	0.93	0.37
17:0	0.63	**0.00**	0.87	0.07	0.78	**0.01**
18:0	0.80	**0.01**	0.76	**0.00**	0.93	0.36
20:0	0.96	0.74	0.89	0.12	0.86	0.05
16:1n7	0.86	0.05	0.76	**0.00**	0.74	**0.00**
17:1n7	0.34	**0.00**	0.52	**0.00**	0.85	**0.04**
18:1n9c	0.63	**0.00**	0.85	**0.03**	0.73	**0.00**
20:1	0.86	0.05	0.93	0.42	0.83	**0.02**
22:1n9	0.93	0.38	0.91	0.23	0.86	**0.04**
24:1n9	0.87	0.07	0.82	**0.02**	0.77	**0.00**
18:2n6c	0.87	0.06	0.90	0.16	0.87	0.07
20:2	0.84	**0.02**	0.90	0.14	0.82	**0.02**
20:3n3	0.89	0.12	0.88	0.09	0.78	**0.01**
20:4n6	0.85	**0.04**	0.93	0.43	0.87	0.07
22:2n6	0.90	0.14	0.92	0.29	0.85	**0.04**
20:5n3	0.94	0.53	0.92	0.27	0.86	0.05
22:6n3	0.80	**0.01**	0.94	0.53	0.81	**0.01**

P.value highlighted in bold indicates significant differences ($P<0.05$).

Supplementary Table 9. Results of tests for significant differences in fatty acids between paired tissues for *Dosidicus gigas* at maturity stage III.

Ovary vs. Mantle			Ovary vs. Digestive gland			Mantle vs. Digestive gland		
FA	statistic	p.value	FA	statistic	p.value	FA	statistic	p.value
Paired t-Tests comparing the FAs satisfying normality			Paired t-Tests comparing the FAs meet normality			Paired t-Tests comparing the FAs satisfying normality		
20:0	-0.77	0.48	14:0	2.32	0.07	16:0	0.20	0.85
22:1n9*	-3.01	0.03	16:0	1.41	0.22	18:0*	4.64	0.01
24:1n9*	-4.40	0.01	17:0	0.00	1.00	20:0*	4.18	0.01
18:2n6c*	-3.17	0.02	20:0*	4.61	0.01	18:2n6c*	4.68	0.01
20:3n3*	-5.38	0.00	20:1*	-4.53	0.01	20:4n6*	3.46	0.02
22:2n6	-0.04	0.97	22:1n9	2.35	0.07	20:5n3*	-3.65	0.01
20:5n3	-0.77	0.47	18:2n6c	3.14	0.05	Paired Wilcoxon tests comparing the FAs not satisfying normality		
Paired Wilcoxon tests comparing the FAs not satisfying normality			20:2	1.31	0:25	14.0*	21.00	0:03
14:0	5.00	0.31	20:3n3*	-11.03	0.00	15:0	20.00	0.06
15:0	15.00	0.44	20:4n6	-2.53	0.05	17:0	20.00	0.06
16:0*	21.00	0.03	22:2n6*	3.61	0.02	16:1n7*	21.00	0.03
17:0	3.00	0.16	20:5n3*	-3.27	0.02	17:1n7*	21.00	0.03
18:0	5.00	0.31	22:6n3	-0.14	0.89	18:1n9c	18.00	0.16
16:1n7	3.00	0.16	Paired Wilcoxon tests comparing the FAs not satisfying normality			20.1	13.00	0:69
17:1n7	4.00	0.86	15:0*	21.00	0.03	22:1n9*	21.00	0.03
18:1n9c	4.00	0.22	18:0	7.00	0.56	24:1n9*	21.00	0.03
20:1*	0.00	0.03	16:1n7	21.00	0.05	20:2*	21.00	0.03
20:2*	1.00	0.04	17:1n7	15.00	0.44	20:3n3	8.00	0.69
20:4n6*	0.00	0.03	18:1n9c	16.00	0.31	22:2n6	12.00	0.84
22:6n3*	21.00	0.03	24:1n9*	21.00	0.03	22:6n3*	0.00	0.03

Asterisk “*” indicates significant differences ($P<0.05$).

Supplementary Table 10. Results of one-sample Kolmogorov-Smirnov tests for each fatty acid content between paired tissues for *Dosidicus gigas* at maturity stage IV.

Fatty acid	Ovary vs. Mantle		Ovary vs. Digestive gland		Mantle vs. digestive gland	
	statistic	p.value	statistic	p.value	statistic	p.value
14:0	0.66	**0.00**	0.92	0.40	0.83	**0.03**
15:0	0.88	0.12	0.64	**0.00**	0.58	**0.00**
16:0	0.77	**0.01**	0.84	**0.04**	0.98	0.97
17:0	0.78	**0.01**	0.88	0.14	0.72	**0.00**
18:0	0.82	**0.02**	0.88	0.12	0.77	**0.01**
20:0	0.84	**0.04**	0.73	**0.00**	0.80	**0.02**
16:1n7	0.90	0.22	0.85	0.06	0.84	**0.04**
17:1n7	0.63	**0.00**	0.87	0.11	0.85	0.05
18:1n9c	0.69	**0.00**	0.91	0.25	0.73	**0.00**
20:1	0.89	0.17	0.88	0.12	0.92	0.39
22:1n9	0.91	0.27	0.93	0.40	0.84	**0.04**
24:1n9	0.88	0.15	0.86	0.08	0.85	0.07
18:2n6c	0.76	**0.01**	0.92	0.34	0.88	0.11
20:2	0.84	0.05	0.70	**0.00**	0.72	**0.00**
20:3n3	0.85	0.06	0.88	0.14	0.78	**0.01**
20:4n6	0.85	0.05	0.96	0.76	0.92	0.35
22:2n6	0.78	**0.01**	0.93	0.45	0.79	**0.01**
20:5n3	0.94	0.50	0.90	0.23	0.95	0.65
22:6n3	0.82	**0.03**	0.89	0.15	0.82	**0.03**

P.value highlighted in bold indicates significant differences ($P<0.05$).

Supplementary Table 11. Results of tests for significant differences in fatty acids between paired tissues for *Dosidicus gigas* at maturity stage IV.

Ovary vs. Mantle			Ovary vs. Digestive gland			Mantle vs. Digestive gland		
Fatty acid	statistic	p.value	Fatty acid	statistic	p.value	Fatty acid	statistic	p.value
Paired t-Tests comparing the FAs satisfying normality			Paired t-Tests comparing the FAs meet normality			Paired t-Tests comparing the FAs satisfying normality		
15:0	0.38	0.72	14:0*	3.51	0.02	16:0	0.02	0.99
16:1n7	-1.47	0.22	17:0	-0.69	0.53	17:1n7*	5.87	0.00
20:1*	-6.77	0.00	18:0	-2.41	0.07	20:1	1.01	0.37
22:1n9*	-7.87	0.00	16:1n7*	4.48	0.01	24:1n9*	6.02	0.00
24:1n9*	-3.56	0.02	17:1n7*	6.26	0.00	18:2n6c*	4.59	0.01
20:3n3	-2.47	0.07	18:1n9c	0.84	0.45	20:4n6*	5.29	0.01
20:4n6*	-3.44	0.03	20:1	-3.20	0.05	20:5n3	-1.28	0.27
20:5n3	-1.89	0.13	22:1n9	1.36	0.25	Paired Wilcoxon tests comparing the FAs not satisfying normality		
Paired Wilcoxon tests comparing the FAs not satisfying normality			24.1n9*	5.18	0:01	14.0	15.00	0:06
14:0	2.00	0.19	18:2n6c*	3.74	0.02	15:0	15.00	0.06
16:0*	15.00	0.04	20:3n3	-3.60	0.05	17:0	5.00	0.63
17:0	1.00	0.13	20:4n6	-1.24	0.28	18:0	14.00	0.13
18:0	0.00	0.06	22:2n6	-0.43	0.69	20:0	12.00	0.31
20:0	5.00	0.63	20:5n3	-2.73	0.05	16:1n7	15.00	0.06
17:1n7	1.00	0.42	22:6n3	-0.39	0.71	18:1n9c	12.00	0.31
18:1n9c	0.00	0.06	Paired Wilcoxon tests comparing the FAs not satisfying normality			22.1n9	15.00	0:06
18:2n6c	3.00	0.31	15:0	13.00	0.19	20:2	15.00	0.06
20:2*	4.00	0.04	16:0	14.00	0.13	20:3n3	10.00	0.63
22:2n6	11.00	0.44	20:0	11.00	0.44	22:2n6	4.00	0.44
22:6n3	15.00	0.06	20:2	12.00	0.31	22:6n3	0.00	0.06

Asterisk “*” indicates significant differences (P<0.05).

Supplementary Table 12. Results of one-sample Kolmogorov-Smirnov tests for each fatty acid content between paired tissues for *Dosidicus gigas* at maturity stage V.

Fatty acid	Ovary vs. Mantle		Ovary vs. Digestive gland		Mantle vs. digestive gland	
	statistic	p.value	statistic	p.value	statistic	p.value
14:0	0.70	**0.00**	0.98	0.94	0.82	**0.03**
15:0	0.71	**0.00**	0.90	0.20	0.80	**0.02**
16:0	0.77	**0.01**	0.88	0.12	0.95	0.69
17:0	0.58	**0.00**	0.66	**0.00**	0.81	**0.02**
18:0	0.85	0.07	0.91	0.30	0.85	0.05
20:0	0.74	**0.00**	0.90	0.24	0.85	0.07
16:1n7	0.69	**0.00**	0.83	**0.03**	0.80	**0.01**
17:1n7	0.64	**0.00**	0.82	**0.03**	0.85	0.06
18:1n9c	0.78	**0.01**	0.89	0.18	0.74	**0.00**
20:1	0.91	0.29	0.90	0.22	0.94	0.58
22:1n9	0.65	**0.00**	0.75	**0.00**	0.66	**0.00**
24:1n9	0.72	**0.00**	0.90	0.20	0.85	0.06
18:2n6c	0.96	0.79	0.92	0.38	0.89	0.19
20:2	0.80	**0.01**	0.92	0.38	0.85	0.06
20:3n3	0.88	0.13	0.85	0.07	0.93	0.49
20:4n6	0.79	**0.01**	0.90	0.23	0.79	**0.01**
22:2n6	0.89	0.19	0.88	0.12	0.87	0.10
20:5n3	0.59	**0.00**	0.73	**0.00**	0.96	0.77
22:6n3	0.63	**0.00**	0.91	0.29	0.86	0.08

P.value highlighted in bold indicates significant differences ($P<0.05$).

Supplementary Table 13. Results of tests for significant differences in fatty acids between paired tissues for *Dosidicus gigas* at maturity stage V.

Ovary vs. Mantle			Ovary vs. Digestive gland			Mantle vs. Digestive gland		
Fatty acid	statistic	p.value	Fatty acid	statistic	p.value	Fatty acid	statistic	p.value
Paired t-Tests comparing the FAs satisfying normality			Paired t-Tests comparing the FAs meet normality			Paired t-Tests comparing the FAs satisfying normality		
18:0	-1.21	0.29	14:0	0.65	0.55	16:0	0.36	0.74
20:1	-2.10	0.10	15:0	1.26	0.28	18:0*	4.00	0.02
18:2n6c*	-2.45	0.04	16:0*	5.07	0.01	20:0*	3.76	0.02
20:3n3	-1.82	0.14	18:0	0.43	0.69	17:1n7*	6.53	0.00
22:2n6*	3.29	0.03	20:0	1.83	0.14	20:1	-0.52	0.63
Paired Wilcoxon tests comparing the FAs not satisfying normality			C18.1n9c	1.32	0:26	24.1n9*	5.48	0:01
14:0	1.00	0.13	20:1	-1.92	0.13	18:2n6c*	5.23	0.01
15:0	7.00	1.00	24:1n9*	3.53	0.02	20:2*	4.87	0.01
16:0	15.00	0.06	18:2n6c*	4.25	0.01	20:3n3	-0.75	0.50
17:0*	4.00	0.04	20:2	1.81	0.14	22:2n6	-2.72	0.05
20:0	9.00	0.81	20:3n3	-2.43	0.07	20:5n3	-1.12	0.33
16:1n7	1.00	0.13	20:4n6	-0.93	0.40	22:6n3*	-4.71	0.01
17:1n7	5.00	0.42	22:2n6*	2.80	0.04	Paired Wilcoxon tests comparing the FAs not satisfying normality		
18:1n9c	2.00	0.19	22:6n3	-0.97	0.38	14:0	15.00	0.06
22:1n9	3.00	0.31	Paired Wilcoxon tests comparing the FAs not satisfying normality			15.0	14.00	0:13
24:1n9	1.00	0.13	17:0	5.00	0.63	17:0	15.00	0.06
20:2*	4.00	0.04	16:1n7	15.00	0.06	16:1n7	15.00	0.06
20:4n6*	5.00	0.04	17:1n7	15.00	0.06	18:1n9c	12.00	0.31
20:5n3	0.00	0.06	22:1n9	10.00	0.63	22:1n9	15.00	0.06
22:6n3	15.00	0.06	20:5n3	1.00	0.13	20:4n6	14.00	0.13

Asterisk “*” indicates significant differences (P<0.05).
